# Hip stress distribution - Predictor of dislocation in hip arthroplasties. A retrospective study of 149 arthroplasties

**DOI:** 10.1371/journal.pone.0225459

**Published:** 2019-11-20

**Authors:** Matevž Tomaževič, Tina Kaiba, Urban Kurent, Rihard Trebše, Matej Cimerman, Veronika Kralj-Iglič

**Affiliations:** 1 Laboratory of Clinical Biophysics, Faculty of Health Sciences, University of Ljubljana, Ljubljana, Slovenia; 2 Department of Traumatology, Division of Surgery, University Medical Centre Ljubljana, Ljubljana, Slovenia; 3 Orthopaedic Hospital Valdoltra, Ankaran, Slovenia; Consorci Parc de Salut MAR de Barcelona, SPAIN

## Abstract

Dislocation after hip arthroplasty is still a major concern. Recent study of the volumetric wear of the cup has suggested that stresses studied in a one-legged stance model could predispose arthroplasty dislocation. The aim of this work was to study whether biomechanical parameters of contact stress distribution in total hip arthroplasty during a neutral hip position can predict a higher possibility of the arthroplasty dislocating. Biomechanical parameters were determined using 3-dimensional mathematical models of the one-legged stance within the HIPSTRESS method. Geometrical parameters were measured from standard anteroposterior X-ray images of the pelvis and proximal femora. Fifty-five patients subjected to total hip arthroplasty that later suffered dislocation of the head and, for comparison, ninety-four total hip arthroplasties that were functional at least 10 years after the implantation, were included in the study. Arthroplasties that suffered dislocation had on average a 6% higher resultant hip force than the control group (p = 0.004), 11% higher peak stress on the load-bearing area (p = 0.001) and a 50% more laterally positioned stress pole (p = 0.026), all parameters being less favorable in the group of unstable arthroplasties. There was no statistically significant difference in the gradient index or in the functional angle of the weight bearing. Our study showed that arthroplasties that show a tendency to push the head out of the cup in the representative body position—the one-legged stance—are prone to dislocation. An unfavorable resultant hip force, peak stress on the load bearing and laterally positioned stress pole are predictors of arthroplasty dislocation.

## Introduction

Based on patient reported outcome measures, hip arthroplasty is the most successful elective surgical procedure [1]. Thanks to efforts to design and produce an optimal prosthesis offering long-term functionality, more than 95% of arthroplasties survive more than 10 years [2,3]. Some total hip arthroplasties (THAs) nevertheless fail and revision surgery is needed. Hip dislocation after THA is the second most common cause of revision surgery [2]. The reported rate of revision due to dislocation after primary THA is 2–4% in the first six months [4] and increases to 6% after 20 years [5]. After revision surgery, the dislocation rate levels at 5.4% in the first year after the operation [6]. Dislocation studies of THA have been performed based on component positioning, with an emphasis on cup orientation [7–10], the effect of artificial head size [5,11,12] and impingement as causes of a prosthesis head dislocation [13–15]. Still more than 50% of dislocations occur when the cup is in the so-called safe zone position [10]. One of the causative factors for instability after THA might be spino-pelvic imbalance [16], decreased mobility of the spine, decreased tilt of the pelvis that might cause impingement [17] and liner wearing [18]. According to latest review soft tissue insufficiency might be the cause for dislocations and not the approach used [19]. A larger femoral head diameter increases the range of motion before the prosthesis neck impinges on the acetabulum liner, which causes the prosthesis to dislocate [5,11,20]. Despite the careful positioning and selection of THA components, dislocations still occur. The question arises as to whether THA changes the geometry of the hip in such a way as to affect the biomechanical parameters in the joint, forcing the hip to dislocate at the edge of the motion range.

The HIPSTRESS method was developed to calculate biomechanical parameters in the hip considering pelvis and femur anatomy [21,22]. Using this method, the hip stress distribution can be calculated in a neutral hip position during a one-legged stance, using a standard X-ray image of the hip and pelvis. It has been validated by clinical studies considering various pathologies in native hips [23,24] and in hips with total hip arthroplasties [25–27].

The HIPSTRESS method demonstrates that linear wear occurs in the direction of the stress pole [25,26] and that it is proportional to the peak stress on the weight bearing area [25]. Because of this effect, the volumetric wear on the cup is less for a larger abduction angle of the cup [27], since the head partly migrates out of the socket [27]. On the other hand, it has been shown that this could be unfavorable in terms of dislocation [27]. The aim of this work was to provide an answer to the question posed by the results of previous work [27]: whether arthroplasties that have suffered dislocation have a less favorable stress distribution.

The hypothesis of this work was that biomechanical parameters (higher peak stress, more lateral stress pole and less negative stress gradient index) are predictors for dislocation of a THA. To test this hypothesis, we compared the biomechanical parameters of a population of THA that had suffered dislocation and a population of THA that were successful at least 10 years post-operatively.

## Methods

The study was designed as a retrospective individual case control study, level of evidence 3B. It was approved by Slovene National Medical Ethics Committee letter No.: 110/04/15 which serves also as an institutional review board approval prior to performing the study. Since the study was retrograde and biomechanical analysis was done on the X-ray images that were coded and already taken before the beginning of the study no informed consent was needed.

Anteroposterior (AP) X-ray images of the hip and pelvic skeleton of patients that had undergone THA were used to measure geometrical parameters relevant for a determination of biomechanical parameters within the HIPSTRESS method. X-ray images of patients that had suffered dislocation of hip arthroplasties were included in the study group. Patients were chosen from the emergency department database on the basis of a diagnosis of hip dislocation, ICD S73.0. Patients admitted to the Emergency Department, University Clinical Center Ljubljana from November 2012 until September 2015 were included. Images were downloaded from the Impax server and coded. Eighty-one patients with a diagnosis of dislocation of the hip joint were gathered. Exclusion criteria were patients that had suffered hip dislocation due to high energy trauma (17 patients), patients with whom dislocation had occurred due to material breakage of the hip prosthesis (2 patients) and patients with whom the contours of the femur or pelvis were not clearly visible on the X-ray image (1 patient). X-ray images of the whole pelvis and hips after reduction of the dislocation were used for analysis. Images were taken in a supine position. The patients were awake when taking the image. After excluding all patients with the exclusion criteria, 61 patients remained in the study group. Forty-one of them were female and 20 male, and the average age was 64.4 years. There were 24 (39%) right hips and 37 (61%) left hips. Twenty-five (41%) of them had already undergone hip arthroplasty on the contralateral hip. The time from the operation to dislocation was on average 402,1 (SD 771,4) days. Dislocation occurred in 47 (77%) cases in less than one year after the THA procedure. The average time to dislocation in this group was 57,2 (SD 79,7) days. Dislocations occurred between 1 and 10 years in 14 (23%) cases after the THA procedure (average 4,27 years with SD 2.53 years). In the study group, the posterior approach was used in 9 patients, the anterolateral approach in 12 patients and the lateral approach in 40 patients.

Patients with partial arthroplasty involving two artificial femoral heads (6 patients) were excluded from the study, since different biomechanics applies due to the double mobility of the partial arthroplasty joint [28].

Patients admitted with partial hip arthroplasties and three patients with THA (altogether 9 patients) were operated at the Clinical Department of Traumatology, University Medical Center Ljubljana. In these patients, the size of the femoral head could be retrieved from the archive. In the remaining 52 patients, the size of the femoral head was not known. Standard X-ray images of the hip and pelvis taken at the Emergency Department, University Medical Center Ljubljana were assumed to have an average magnification of 115% and the size of the prosthesis head was estimated by rounding to 28mm, 32mm or 36 mm using software developed for preoperative planning at the Department of Traumatology, University Medical Center Ljubljana. Twenty-six (47%) hips in the study group with THA had a femoral head diameter of 28 mm, 12 (22%) had a femoral head diameter of 32 mm and 17 (31%) had a femoral head diameter of 36 mm. On average, the radius of the prosthesis head was 15.67 mm ± 1.75 mm. In the study group, there were two cases where elevated liners were seen on the x ray pictures. The data on the inlay and the prosthesis head were available from the archive for three cases only. In these three cases we did not see any elevated liner. Stems were cemented in 26 cases and uncemented in 29 cases. None of the femoral stems were loosened. The acetabulum components were uncemented in 23 cases and cemented in 32 cases. There were signs of loosening of the cemented acetabulum component in one case.

To define the control group, we examined X-ray images of 311 patients who had undergone total hip arthroplasty (THA) at the Orthopedic Hospital Valdoltra, Slovenia. The first available X-ray images or THA with whole pelvis (in terms of date) were taken from the Impax server at the hospital. Inclusion criteria were an X-ray image of the pelvis and proximal femora after implantation, no event of hip dislocation or septic or aseptic loosening and regular follow-up for at least 10 years. Patients who had undergone any revision procedure during that time were also excluded. Ninety-four hips (54 (63%) right and 35 (37%) left) of 77 patients that met the inclusion criteria (45 female and 32 male) were included in the analysis. Sixty-nine of them also had a prosthesis on the contralateral side. The average age of the patients included at the time of implantation was 59.6 years. There were 79 (84%) THA with a femoral head diameter of 28mm, 2 (2%) of them had a femoral head diameter of 32 mm and 13 (14%) had a femoral head diameter of 36mm. In the control group, acetabulum was cemented in 7 cases and uncemented in 87 cases. Inlay was ceramic–alumina in 20 cases and polyethylene in 60 cases (UHMWPE in 44 cases, crosslinked PE in 14 cases and Eduron PE in 2 cases). Head was ceramic–alumina in 34 cases, CoCr alloy in 30 cases and Fe alloy in 30 cases. Femur stem was cemented in 5 cases and uncemented with diaphyseal fit in 89 cases. In the control group, lateral approach was used in all THA procedures.

**Fig 1:**
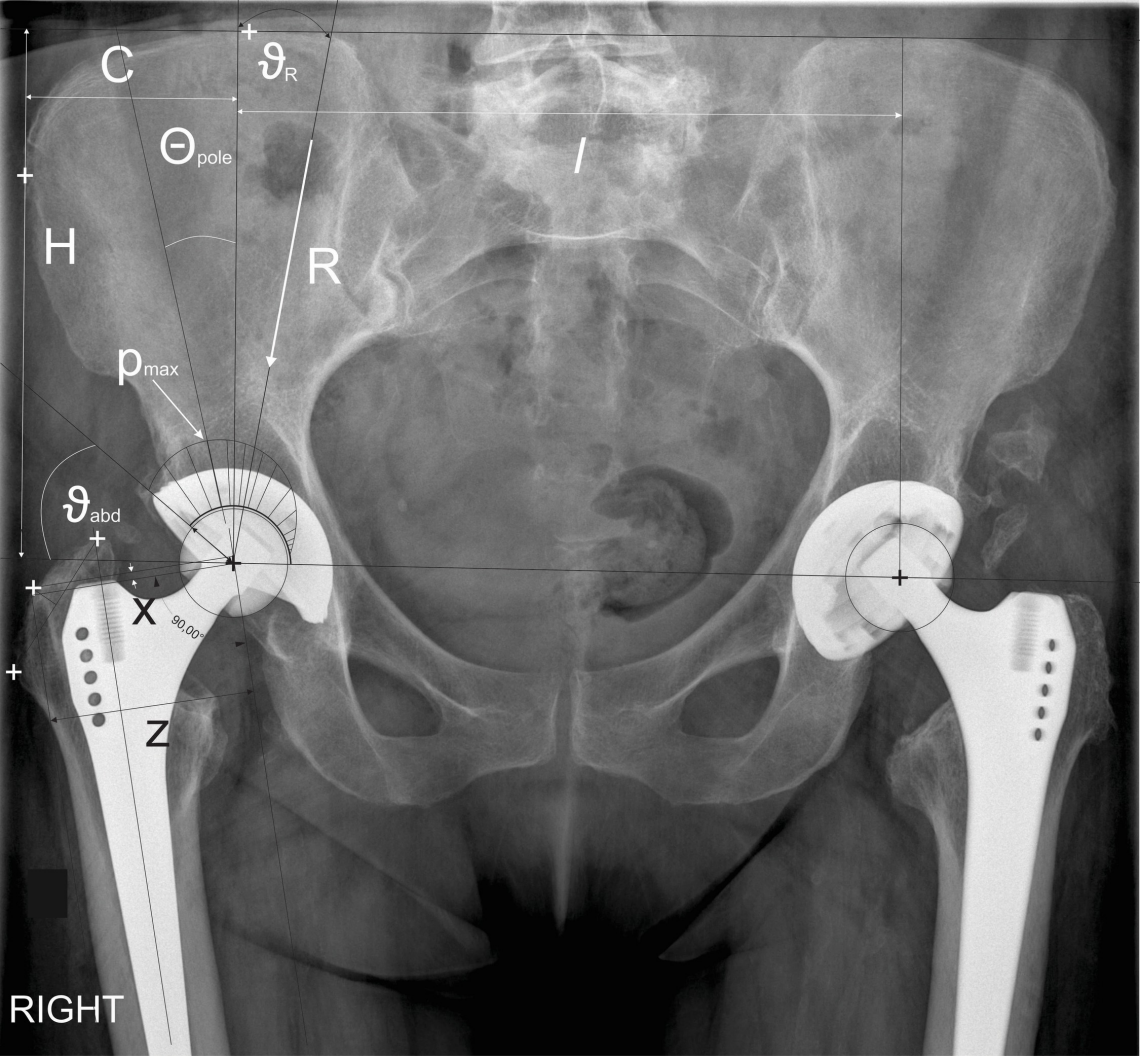
Geometric and biomechanical parameters within the HIPSTRESS models that are used for calculation of stress distribution in a right artificial hip. *R* resultant hip force; *p*_max_ peak stress on the load bearing area; θ_pole_ angle of the stress pole; ϑ_abd_ abduction angle; ϑ_*R*_ angle of the resultant hip force; *C* horizontal distance between the center of the prosthesis head and the most lateral point on the iliac crest; *H* vertical distance between the center of the prosthesis head and the highest point on the iliac crest; *x* vertical distance between the center of the prosthesis head and the point on the greater trochanter in the direction of the femur; *z* distance between the center of the prosthesis head and the point on the greater trochanter perpendicular to the femur axis; *l* distance between the centers of the femoral heads.

For biomechanical evaluation, three-dimensional mathematical models of an adult human hip within the HIPSTRESS method were used [22,24]. The models are described in detail elsewhere (see for example ref. [24]) so only a brief description will be given here. The method consists of two mathematical models: one for determination of the resultant hip force in the representative body position for everyday activities [29], i.e., the one-legged stance [30], and the other for determination of contact hip stress distribution [21]. The model for resultant hip force is based on force and torque equilibrium equations [22,30]. The model describes a system composed of two segments: the loaded leg and the rest of the body. It includes 9 effective muscle forces, the weight of the segments and the intersegment force (the resultant hip force). The reference muscle attachment points are obtained from measurements performed on a cadaver and then re-scaled for the individual hip considered. Since the X-ray image is two-dimensional, data in the third dimension are taken to be equal to the reference values. The model for force uses as input the geometrical parameters of pelvis and proximal femur: pelvic width (*C*) and height (*H*), inter-hip distance (*l*) and the position of the muscle attachment point on the greater trochanter (*x*,*z*) (Fig 1). Stress integrated over the load-bearing area yields the resultant hip force R = ʃ *p* dA, where *p* is stress and dA is the area element. The calculations and procedures have been explained previously [23–25,31].

HIPSTRESS models use hip and pelvis geometric parameters as input data: inter-hip distance (*l*), height of the pelvis (*H*), horizontal distance from the prosthesis head center to the lateral edge of the pelvis (*C*), position of the greater trochanter relative to the prosthesis head center in the coordinate system of the femur (distances *z* and *x*) and abduction angle. The geometrical parameters were determined using CorelDRAW Graphics Suite X7, 2015, Ottawa, Canada, by two blinded measurers. HIPSTRESS software [31] was used for calculation of the biomechanical parameters. (Fig 1)

Previous studies [32–36] have indicated that the peak stress on the load-bearing area *p*_max_ is a useful biomechanical parameter_._ If the stress pole is located inside the load bearing area, *p*_max_ is equal to the value of the stress at the pole. If the stress pole lies outside the load-bearing area, contact stress is highest at the point of the load-bearing area that is closest to the pole (Eq 1),

$$p_{\max}=p_{0}\cos\left(\frac{\pi }{2}-\vartheta_{abd}-\Theta_{pole}\right)$$ (1)

where *p*_0_ is the value of stress at its pole, ϑ_abd_ is the abduction angle of the prosthesis cup and Θ_pole_ is the angle of the stress pole (Fig 1). Another indicator of the stress distribution is the gradient of stress, represented by its value at the lateral rim of the cup *G*_p_ (Eq 2) [27,37],

$$G_{p}=p_{0}\cos(\pi -\vartheta_{abd}-\Theta_{pole})/r$$ (2)

where *r* is the radius of the articular surface. If the pole of stress distribution lies outside the load-bearing area (i.e., if Θ_pole_ > π/2 –ϑ_abd_), then *G*_*p*_ is positive, stress attains its highest value at the lateral rim and falls off rapidly in the medial direction, while the corresponding weight bearing area is small [38]. Such a distribution represents dysplastic hips [38]. If, however, the pole of stress distribution lies inside the load–bearing area (if Θ_pole_ < π/2 –ϑ_abd_), then stress reaches its peak within the weight bearing area, which is consequently larger and *G*_*p*_ is negative [38]. The functional angle of load bearing ϑ_f_ is defined as (Eq 3).

$$\vartheta_{f}=(\pi -\vartheta_{abd}-\Theta_{pole})$$ (3)

In relation to dislocation, high resultant hip force and high peak stress are unfavorable. However, a high gradient index and more laterally positioned pole are expected to represent an even greater risk of dislocation, since the head is pushed more laterally each time the leg is loaded.

The peak stress *p*_max_ is proportional to *r*^-2^, while the hip stress gradient index *G*_*p*_ is proportional to *r*^-3^ [21,38]. Since different sizes of prosthesis heads were involved, the effect of the femoral head size on biomechanical parameters was eliminated by multiplying *p*_max_ by *r*^2^ and *G*_*p*_ by *r*^3^. The resultant hip force, peak stress and stress gradient index are also proportional to body weight *W*_b_. Since the body weight was unknown, its effect was eliminated by normalizing the respective parameters by *W*_b_. The normalized biomechanical parameters *R*/*W*_B_, *p*_max_*r*^2^/*W*_b_, *G*_*p*_*r*^3^/*W*_b_, ϑ_f_ and Θ_pole_ express the geometry of the pelvis and the proximal femur and the geometry and position of the arthroplasty’s elements (but not the size of the artificial head).

Statistical analysis of the biomechanical parameters between the two groups was done using the *Student T*—test. If the p value was ≤ 0.05, the difference between these two groups was significant. The statistical power (1-β) of the result was taken as sufficient when the power was >80%. Statistical analysis was done using Microsoft Excel 2010 (14.0.7188.5000, Microsoft Corporation, Santa Rosa, California, USA). The power of the statistics was calculated using a statistics power calculator on the internet: http://clincalc.com/Stats/Power.aspx

## Results

Normalized resultant hip force *R/W*_b_ and normalized peak stress *p*_max._*r*^2^/*W*_b_ were considerably and statistically significantly less favorable in the study group than in the control group, with sufficient statistical power. The position of the stress pole was more lateral (less favorable) in the study group. The difference was statistically significant but with somewhat deficient statistical power (Table 1). The normalized stress gradient index *G*_*p*._*r*^3^/*W*_b_ and functional angle of load bearing showed no statistically significant difference, although it was less negative and smaller (which is unfavorable) in the study group.

**Table 1:** Comparison of biomechanical parameters of the hips with THA in the study and control group.

Average ± SD	Study group (55 THA)	Control group (94 THA)	Difference (%)	p	Power (1-β)
*R*/*W*_b_	mean 2.71 (SD 0.36)*	mean 2.54 (SD 0.32)	6.3	0.004	89.9%
*p*_max_ x *r*^2^/*W*_b_	mean 152.11 (SD 37.04)*	mean 135.32 (SD 21.42)	11.0	0.001	92.7%
*G*_*p*_ x *r*^3^/*W*_b_	mean -828287.70 (SD 413381.01)*	mean -871926.04 (SD 220240.41)	-5.27	0.400	10.9%
ϑ_f_ (°)	mean 129.13 (SD 17.57)	mean 132.90 (SD 13.60)	-2.9	0.146	39.5%
θ_pole_ (°)	mean 5.77 (SD 9.71)*	mean 2.86 (SD6.08)	50.0	0.026	62.9%

*R/W*_b_, resultant hip force normalized by body weight; *p*_max_ x *r*^2^/*W*_b_; effect of pelvis geometry on peak stress on the load bearing area normalized by body weight; *G*_*p*_ x *r*^3^/*W*_b_; effect of pelvic geometry on the peak hip gradient index; ϑ_f_ (°), functional angle of the load bearing; θ_pole_ (°), position of the stress pole.

(*) An asterisk denotes a value with higher risk for dislocation

There was a considerable and statistically significant difference in parameters *x* (distance between the center of the prosthesis head and the point on the greater trochanter in the direction of the femur axis) and *C* (horizontal distance between the center of the prosthesis head and the most lateral point of the iliac crest) between the study and control groups (Table 2). Higher *x* and *C* indicate less favorable biomechanical parameters in the study group. The difference between the abduction angles in the study group and control group was minute, which explains the lack of statistical significance of the difference in the functional angle of weight bearing (Table 1).

**Table 2:** Comparison of geometrical parameters of the hips with THA in the study group and control group.

Average ± SD	Study group (55 THA)	Control group (94 THA)	Difference (%)	p	Power (1-β)
*H* (mm)	mean 136.80 (SD 13.53)	mean 133.78 (SD11.18)	2.2	0.144	40.6%
*z* (mm)	mean 56.73 (SD 8.71)	mean 59.18 (SD 6.73)	-4.3	0.057	56.3%
*x* (mm)	mean 14.77 (SD 8.69)*	mean 7.81 (SD 5.34)	47.1	0.000	100%
*C* (mm)	mean 59.58 (SD 8.87)*	mean 55.52 (SD 9.22)	6.8	0.010	84.8%
*l* (mm)	mean 179.61 (SD 11.15)	mean 177.13 (SD 10.26)	1.38	0.169	10.9%
ϑ_abd_ (°)	mean 45.10 (SD9.12)	mean 44.24 (SD 7.97)	1.9	0.547	14.5%

*H* (mm), vertical distance between the center of the prosthesis head and the highest point on the iliac crest; *z* (mm), distance between the center of the prosthesis head and the point on the greater trochanter perpendicular to the femur axis; *x* (mm), vertical distance between the center of the prosthesis head and the point on the greater trochanter in the direction of the femur; *C* (mm), horizontal distance between the center of the prosthesis head and the most lateral point on the iliac crest; *l* (mm), horizontal distance between the right and left center of the femoral head; ϑ_abd_ (°), abduction angle. (*) An asterisk denotes a value with a higher risk of dislocation.

To evaluate whether there were any significant differences in the geometry of the pelvis and hip biomechanics between the study and control groups, a comparison of the native hips contralateral to the arthroplasties between the study and control groups was performed. Since there were cases in both groups with bilateral arthroplasties, only subjects without contralateral hip arthroplasty were taken for comparison.

There were no statistically significant differences between hips contralateral to arthroplasties that had suffered dislocation and arthroplasties that were functional at least 10 years post-operatively (Tables 3 and 4). This indicates that the geometry of the pelvis and proximal femur of the patient before arthroplasty is not the cause of the difference between the study and control groups and that changes induced by arthroplasty surgery are connected to the difference in the biomechanical parameters of the study and control groups.

**Table 3:** Comparison of biomechanical parameters of native hips contralateral to arthroplasties in the study and control group.

Average ± SD	Native hips contralateral to arthroplasties in the study group (36 hips)	Native hips contralateral to arthroplasties in the control group (25 hips)	Difference (%)	p	Power (1-β)
*R/W*_b_	mean 2.71 (SD 0.89)	mean 2.66 (SD 0.42)	1.76	0.780	8.8%
*p*_max_ x *r*^2^/*W*_b_	mean 168.77 (SD 65.97)	mean 183.27 (SD 87.25)	8.59	0.485	17.3%
*G*_*p*_ x *r*^3^/*W*_b_	mean -480308.69 (SD 730010.97)	mean -561387.71 (SD 341552.74)	14.44	0.605	14.4%
ϑ_f_ (°)	mean 113.69 (SD 15.86)	mean 113.43 (SD1 9.16)	0.23	0.955	5.1%
θ_pole_ (°)	mean 11.16 (SD7.44)	mean 12.50 (SD 11.06)	11.96	0.601	13.2%

*R/W*_b_, resultant hip force normalized by body weight; *p*_max_ x *r*^2^/*W*_b_; effect of pelvis geometry on peak stress on the load bearing area normalized by body weight; *G*_*p*_ x *r*^3^/*W*_b_; effect of pelvic geometry on the peak hip gradient index; ϑ_f_ (°), functional angle of the load bearing; θ_pole_ (°), position of the stress pole.

**Table 4:** Comparison of geometrical parameters of native hips contralateral to arthroplasties in the study and control group.

Average ± SD	Native hips contralateral to arthroplasties in the study group (36 hips)	Native hips contralateral to arthroplasties in the control group (25 hips)	Difference (%)	P	Power (1-β)
*H* (mm)	mean 139.19 (SD 10.65)	mean 142.05 (SD 13.89)	2.06	0.389	21.8%
*z* (mm)	mean 60.25 (SD 5.62)	mean 58.24 (SD 7.93)	3.34	0.279	29%
*x* (mm)	mean 6.33 (SD 5.09)	mean 7.61 (SD 4.81)	20.17	0.324	25.9%
*C* (mm)	mean 52.88 (SD 9.83)	mean 56.61 (SD 10.42)	7.07	0.164	40.6%
*x* (mm)	mean 6.33 (SD 5.09)	mean 7.61 (SD 4.81)	20.17	0.324	25.9%
*l*(mm)	mean 181.28 (SD 14.07)	mean 177.74 (SD 10.13)	2	0.279	20.7%
ϑ_abd_(°)	mean 55.15 (SD8.92)	mean 54.07 (SD 9.70)	-3.08	0.662	11.4%

*H* (mm), vertical distance between the center of the prosthesis head and the highest point on the iliac crest; *z* (mm), distance between the center of the prosthesis head and the point on the greater trochanter perpendicular to the femur axis; *x* (mm), vertical distance between the center of the prosthesis head and the point on the greater trochanter in the direction of the femur; *C* (mm), horizontal distance between the center of the prosthesis head and the most lateral point on the iliac crest; *l* (mm), horizontal distance between the right and left center of the femoral head; ϑ_abd_ (°), abduction angle.

## Discussion

Our study supports the hypothesis that contact stress distribution on the prosthesis head is a predictor of arthroplasty dislocation. The hypothesis of this study was formed following previous work by Rijavec et al. [27], in which the effect of cup inclination on predicted contact stress-induced volumetric wear in THA was considered. In that study, it was indicated that a larger abduction angle of the prosthesis cup is more favorable in terms of prosthesis wear, although it can represent a larger risk of its dislocation.

It is shown above (Table 1) that THA that had suffered dislocation had a less favorable distribution of contact stress (given by its peak value and the position of the pole), which pushed the artificial head more laterally than in the case of prostheses that were functional at least 10 years post-operatively. A previous study had shown that the head migrates in the direction of the stress pole [25]. This process changes the shape of the interface between the head and the cup and contributes to the development of a lever that leads to dislocation.

Comparison of native hips contralateral to dislocated and functional arthroplasties showed no statistically significant differences, in either biomechanical parameters or geometrical parameters (Tables 3 and 4), indicating that the initial geometry of the pelvis and proximal femur was on average the same in the two groups. It is the change in pelvic anatomy after the arthroplasty procedure that changes the biomechanics of the hip and these changes were less favorable in the group of prostheses that had suffered dislocation.

Dislocation of the hip joint after THA is one of the major side complications (2% - 4% after primary total hip arthroplasty) [2,39] and its causes have been studied previously [5,7–20,39–42]. Among patient related factors, the presence of neuromuscular conditions, such as cerebral palsy, muscle dystrophy, dementia and Parkinson’s disease, is one of the main risk factors [39,43]. More dislocations were found with patients older than 80 years [43], patients with a femoral neck fracture as the primary diagnosis [40,42] and patients with an ASA (American Society of Anesthesiologists) score of 3 or 4 [40]. Among procedure related factors, the measured parameters of component positions, a cup inclination out of the range of 40° ± 10°, a cup anteversion of less than 10° or more than 35°, a stem anteversion out of the range of 14.8° ± 6,01° and a height of hip rotation center outside the range of 2.16 mm ± 9.11 mm, increased the risk of dislocation [8,9,40]. The artificial head size, leg length discrepancy and acetabular inclination were all studied in two separate studies and it was found that these are not statistically important factors predicting dislocation of the femoral head [7,44]. On the other hand, in a study by Berry [5], a smaller size of the prosthesis head was shown to be related to a higher dislocation rate of THA. In a study by Forde et al. [7], it was reported that if the femoral offset was at least 3 mm greater than on the contralateral side, the risk of dislocation was lower [7]. Offset is expressed in the HIPSTRESS model by parameter z. A larger z is biomechanically favorable, since it implies a lower resultant force and larger angle of inclination, which consequently means lower peak stress, a more medial location of the pole, a smaller gradient index and a larger functional angle of weight bearing[24]. Our results are therefore in agreement.

In a study by Rijavec [27] that considered the effect of cup inclination on predicted contact stress-induced volumetric wear in THA, stress distribution was proposed as a relevant factor connected to the probability of dislocation of the artificial head. Our results show that normalized stress was indeed considerably and statistically significantly less favorable in the study group. The position of the pole was statistically significantly less favorable in the study group, while the difference in gradient index *G*_*p*_.*r*^3^/*W*_b_, albeit showing a less favorable configuration in the study group, was not statistically significant. Since three of the parameters were on average less favorable in the study group, our results support the proposed hypothesis.

The most frequently considered measurements in prostheses are abduction angle and the lateral offset [7,44]. We found on average no statistically significant differences between abduction angles in the study and control groups, although the standard deviation was somewhat larger in the study group. The average values of the abduction angle were close to ideal (45 degrees) in both groups, so the differences in biomechanical parameters could not have been due to the abduction angle, which was also found in a study by Forde[7]. On the other hand, the offset is connected to the shape of the greater trochanter. We found that there was a considerable and statistically significant difference in the position of the characteristic point on the greater trochanter, which was less favorable in hips that had suffered dislocation.

Our study and control groups had a different distribution of artificial head sizes and we focused on the effect of the geometry of the pelvis and the proximal femur and the inclination of the artificial cup. We therefore chose biomechanical parameters that were independent of the artificial head size. The effect of femoral head size was considered [45], who reported that a larger head diminishes the probability of impingement between the femoral neck and acetabular liner. Our analysis focused on the one-legged stance and did not consider events connected specifically to other body positions that could predispose the femoral head to dislocate.

Preoperative planning is advised before implantation of an artificial hip and is usually done [46]. Optimization of the choice and position of prosthesis elements using simulation with a mathematical model could be included in preoperative planning. Simulation of postoperative biomechanical parameters would be useful in planning the configuration of prostheses, especially in demographic groups in which hips are more vulnerable to dislocation, or in revision cases. In cases in which preoperative planning predicted an arthroplasty prone to dislocation, the surgeon could decide on another implant configuration.

The main limitation of the study is that biomechanical parameters are determined using mathematical 3D model on the two dimensional AP image of the pelvis and proximal femora. From this image spinopelvic alignment and anteversion of the cup cannot be accessed. In the recent studies [16,17] spinopelvic alignment was suggested as an important factor that could predispose late dislocation of the total hip endoprosthesis.

Other limitation is that the control X-rays of the pelvis and proximal femora after the dislocation were taken in a supine and not in a standing position.

## Conclusions

Our results showed that the shape of the pelvis and proximal femur after total hip arthroplasty impacted on a less favorable stress distribution in the representative everyday activity, the one-legged stance, in prostheses that had suffered dislocation. Although the hip does not dislocate during the one-legged stance, these hips had higher stress, accumulated more laterally, than arthroplasties that were functional for at least 10 years. The more lateral position of the stress pole could remodel the joint and predispose dislocation during other activities.

## Supporting information

S1 File Microsoft Office Excel file with measurements and calculations of all the values. (XLSX)
